# How Knowledge Worker Teams Deal Effectively with Task Uncertainty: The Impact of Transformational Leadership and Group Development

**DOI:** 10.3389/fpsyg.2017.01339

**Published:** 2017-08-15

**Authors:** Jan-Paul Leuteritz, José Navarro, Rita Berger

**Affiliations:** ^1^Human Factors Engineering, Fraunhofer-Institute for Industrial Engineering (IAO) Stuttgart, Germany; ^2^Department of Social Psychology, Universitat de Barcelona Barcelona, Spain

**Keywords:** transformational leadership, group development, task uncertainty, knowledge work, team effectiveness

## Abstract

The purpose of this paper is to clarify how leadership is able to improve team effectiveness, by means of its influence on group processes (i.e., increasing group development) and on the group task (i.e., decreasing task uncertainty). Four hundred and eight members of 107 teams in a German research and development (R&D) organization completed a web-based survey; they provided measures of transformational leadership, group development, 2 aspects of task uncertainty, task interdependence, and team effectiveness. In 54 of these teams, the leaders answered a web-based survey on team effectiveness. We tested the model with the data from team members, using structural equations modeling. Group development and a task uncertainty measurement that refers to unstable demands from outside the team partially mediate the effect of transformational leadership on team effectiveness in R&D organizations (*p* < 0.05). Although transformational leaders reduce unclarity of goals (*p* < 0.05), this seems not to contribute to team effectiveness. The data provided by the leaders was used to assess common source bias, which did not affect the interpretability of the results. Limitations include cross-sectional data and a lower than expected variance of task uncertainty across different job types. This paper contributes to understanding how knowledge worker teams deal effectively with task uncertainty and confirms the importance of group development in this context. This is the first study to examine the effects of transformational leadership and team processes on team effectiveness considering the task characteristics uncertainty and interdependence.

## Introduction

Uncertainty is growing in modern working contexts. Polyvalence, time pressure, unpredictable environmental conditions, and the relevance of knowledge and distributed skills drive this development (Navarro et al., 2011). Knowledge workers are particularly exposed to uncertain tasks and the relevance of knowledge work is rising in the developed economies (Spath and Hofmann, 2009): today's organizations need to constantly innovate (Reuveni and Vashdi, 2015), and they increasingly rely on teams for this purpose (Edmondson and Nembhard, 2009). Consequently, organizations need to enable their teams to deal with uncertainty and to create the synergies necessary to innovate. Although, the literature on leadership is extensive, the role of leadership with respect to the demands of increasingly uncertain tasks has not been investigated, yet. We therefore researched the role of leadership with respect to different types of task uncertainty, taking interpersonal and structural coordination mechanisms into account and addressing limitations of previous research. “Innovation is the multi-stage process whereby organizations transform ideas into new/improved products, services or processes” (Baregheh et al., 2009, p. 1334). Creativity is defined as the generation of such ideas (Cheung and Wong, 2011). Thus, innovation requires creativity. Work meant to produce innovation as its primary outcome has been labeled knowledge work (Willke, 1998; Drucker, 1999). Definitions of knowledge work stress the continuous requirement for learning (Willke, 1998; Drucker, 1999), unclear objectives, processes or outcomes (Spath and Hofmann, 2009), or the fact that knowledge is always connected to the unknown and always improvable (Willke, 1998). The common characteristics across these definitions are uncertain objectives, a lack of familiarity with the methods required to achieve the objective, or an unclear connection between method and outcome of the work. This matches the operationalization of task uncertainty by Navarro et al. (2011, p. 19). Knowledge work is characterized by uncertain tasks.

Consequently, two approaches are available to investigate the factors that help teams innovate: (1) examining which factors influence outcomes such as team innovation or team creativity, and (2) exploring which factors increase the effectiveness of teams working on uncertain tasks.

With respect to the first approach, research evidence is available. It indicates that transformational leadership is particularly beneficial to the workers in teams focused on innovation: leaders should serve as role models (idealized influence), communicate a positive vision (inspirational motivation), take care of followers individually (individualized consideration), and encourage them to find their own solutions (intellectual stimulation; Bass et al., 2003). Thereby, they foster individual worker creativity (De Jong and Den Hartog, 2007), individual employees' engagement in idea management (Pundt and Schyns, 2005), as well as group creativity (Jung, 2001; Eisenbeiß, 2009) and team innovation (Paulsen et al., 2009). Research indicates that the positive effect of transformational leadership on team innovation and team creativity is mediated by group processes such as cohesiveness (Eisenbeiß, 2009), team identity (Paulsen et al., 2009), engagement and knowledge sharing (Edmondson and Lei, 2014), or development of shared mental models (Reuveni and Vashdi, 2015). These findings integrate well into what is generally known about the effects of transformational leadership on teams: transformational leadership augments the positive effects of transactional leadership on team performance (Avolio et al., 2009) and group processes such as cohesiveness are mediators of this relationship (Jung and Sosik, 2002; Bass et al., 2003).

However, existing research does not clarify whether transformational leadership plays a special role in teams with high task uncertainty, such as teams of knowledge workers, compared to teams in other types of work. Answering this question requires evidence based on the second approach, which is not available as far as we know. Literature indicates that transformational leadership is more effective when the organizational environment is uncertain (Bass and Riggio, 2006; Felfe, 2006) and the same could apply to uncertain tasks: Frost et al. (2010) assumed that teams of knowledge workers require transformational management solutions. To test these assumptions, we investigated a model of the relationships between transformational leadership, group processes and task uncertainty. In contrast to previous studies, we compared teams across different job types. Like other studies in this field (e.g., Eisenbeiß, 2009; Reuveni and Vashdi, 2015), this research was focused on the team level.

The work presented here is, to our knowledge, the first study to investigate the relationships between transformational leadership, task uncertainty, and team effectiveness. We tested assumptions derived from Frost et al. (2010) and we addressed the limitations of previous studies resulting from the use of homogeneous samples. In the model, we considered both, interpersonal (group development) and structural (task interdependence) coordination mechanisms.

## Theoretical background and research model

### Research model and independent variable: transformational leadership

As argued above, there is exhaustive evidence that transformational leadership has positive effects on team performance (Avolio et al., 2009) and that group processes such as cohesiveness mediate this relationship (Jung and Sosik, 2002; Bass et al., 2003). While prior research relied on Input-Process-Output Models (I-P-O, e.g., West and Hirst, 2003), Input-Mediator-Output-Input (IMOI) Models are the most appropriate choice: I-P-O models assume the mediating variable to be a process, which is inadequate in many cases; in IMOI, it can be an emergent state, too (Ilgen et al., 2005). As longitudinal data was not available, we integrated the aforementioned relationships into an Input-Mediator-Output model and added measures of task uncertainty. In the following paragraphs, we provide the reasoning for the choice of constructs and hypotheses.

### Dependent variable: team effectiveness

To research the relationships between transformational leadership, group processes and task uncertainty, the outcome variable must be applicable to any kind of team, no matter if such team is meant to produce innovation or not. Therefore, we chose team effectiveness (Hackman, 1987) as our outcome variable: a team is considered effective if (1) it meets the success criteria defined by stakeholders, (2) the team members benefit from the outcomes of the team's work, and (3) the team's ability of working together in the future is maintained. As a criterion of team performance, team effectiveness has a long tradition in team research (e.g., Kozlowski and Bell, 2003).

### Mediator: group development

In the majority of reported models, instead of other processes or emergent states, cohesion is considered as the direct predictor of team effectiveness (Jung and Sosik, 2002; Bass et al., 2003). However, the concept of group cohesiveness, the different ways it is measured and how it is used in research has been criticized (Hogg, 1993). Thus, we replaced cohesion by *group development* (GD; Meneses et al., 2008). This construct represents the degree to which a set of people functions as a real team, defined by these characteristics of well-developed groups (Navarro et al., 2015): (1) there are regular personal interrelationships between the members; (2) the members are working or oriented toward shared goals; (3) the members identify with the group; and (4) the group has a high level of coordination. In contrast to group cohesion, GD refers to the group's goals and to the group's coordination, which we considered highly relevant to explaining the effects of leadership on team outcomes as mediated by group processes.

Theory further justifies the assumption that transformational leadership leads to increased group development: transformational leadership is supposed to raise the acceptance of group goals (Podsakoff et al., 1996), which is a requirement of group development (Navarro et al., 2015). Additionally, individual consideration might reduce conflict among the team members and thus positively affect their interpersonal relationships. Finally, individual consideration and intellectual stimulation could make team members feel appreciated and their contributions valued, which may strengthen their identification with the team. Based on this reasoning and literature (Jung and Sosik, 2002; Bass et al., 2003), we set the following hypothesis:

*H1. Group development will at least partially mediate the positive relationship between transformational leadership and team effectiveness, with all variables being positively interrelated*.

Despite its similarities to previous research, this model has, to our knowledge, never been tested.

### The role of task uncertainty

The next step was adding task uncertainty to the model. Based on the literature, it could be mediator or a moderator, depending on its operationalization. Sicotte and Bourgault (2008), for example, reported some dimensions of organizational and environmental uncertainty to directly predict a decrease in team performance, while other dimensions of uncertainty moderated the effects of organizational interventions on team performance. We intended to represent both potential roles in the model by including *new situations* and *unclarity of goals* from the German version of the MITAG instrument. We had previously validated this instrument in a German sample, which had resulted in a reduced set of items and a new factor structure. From the three newly identified factors, we picked new situations and unclarity of goals. For reasons of model parsimony, we disregarded the third factor named *non-routine*, which on a theoretical level was more difficult to relate to the other constructs.

Previous studies (Faraj and Yan, 2009; Gardner et al., 2012) relied on short questionnaires that did not distinguish between different types of uncertainty, although some were limited to specific work settings. We decided to use measurements that are applicable across different job types while specifying subordinate factors of task uncertainty.

### Task uncertainty as a moderator

As task uncertainty is a necessary requirement of knowledge work (Spath and Hofmann, 2009), some uncertain aspects of the team's task cannot be proactively reduced by the team itself. Variables that measure these types of task uncertainty consequently qualify either as independent or as moderator variables. The model by West and Hirst (2003) supports this perspective by restricting task characteristics to the category of input variables.

There is evidence that transformational leadership is more likely to emerge and more effective, when the environment is complex (Felfe, 2006; Wolfram and Mohr, 2009), unstable, uncertain or turbulent (Bass and Riggio, 2006). This means that environmental complexity and uncertainty moderate the relationship between transformational leadership and team outcomes (Wolfram and Mohr, 2009, p. 261). Consequently, uncertainty related to the team's task could also moderate this relationship. This hypothesis is further supported by Frost et al. (2010): they argued that knowledge work requires intrinsic motivation and voluntary contributions, which are fostered by transformational leadership. Consequently, we argue that there should be an interaction effect between transformational leadership and task uncertainty, which represents the characteristics of knowledge work.

If H1 were true, task uncertainty could moderate either the influence of leadership on group processes, or the effect of group processes on team effectiveness. Literature suggests the latter: Navarro et al. (2011, p. 20) argue that the social support and sense-making activities of group-work are particularly beneficial when dealing with diverse, new, incompatible, and ambiguous tasks. And evidence shows that boundary reinforcement, which refers to “sharpening team identity” (Faraj and Yan, 2009, p. 607), and relational resources such as familiarity among team members (Gardner et al., 2012) are more positively related to team performance when task uncertainty is high. Thus, we assumed that task uncertainty would moderate the relationship between GD and team effectiveness.

To represent this type of externally caused task uncertainty, we used the factor new situations from the German version of the MITAG questionnaire, as resulting from our previous validation study. It refers to conflicting or fast changing short-term demands from outside the team. Thus, it is a type of uncertainty that the team cannot avoid proactively. This type of task uncertainty requires performance adaptations, which have been defined as “altering behavior to meet the demands of the environment, an event or a new situation” (Pulakos et al., 2002, p. 615). Team adaptation requires coordination and information sharing (Maynard et al., 2015), which corresponds to the characteristics of well-developed teams, as measured by the GD instrument. So, we hypothesized that well-developed teams adapt more efficiently to such changing short-term demands.

### Controlling for task interdependence

To test the moderating effect of the factor new situations, we had to control for task interdependence. “Team members are task interdependent when they must share materials, information, or expertise in order to achieve the desired performance or output.” (Van der Vegt et al., 2001, p. 52). The commitment to a shared goal, group coordination, and strong interpersonal relationships can be expected to be helpful in interdependent tasks (Mullen and Copper, 1994), even when uncertainty is low. Consequently:

*H2. New situations will moderate the relationship between GD and team effectiveness, while task interdependence will moderate this moderation effect: combinations of low scores on new situations and task interdependence will be associated with weaker relationships between group development and team effectiveness*.

### Task uncertainty as a mediator

However, task uncertainty can be a mediator if team members or leader can actively reduce or increase a certain aspect of task uncertainty. Weiss and Hoegl (2016) hypothesized that increased task uncertainty will be detrimental to team performance. They argued that task uncertainty required more planning and “more frequent non-routine decision-making,” which would occupy additional team resources such as time and effort (p. 15). Such an effect may have led to Tatikonda and Rosenthal (2000) finding higher task uncertainty to be related to higher costs in technology innovation projects.

We chose the factor unclarity of goals from the German MITAG questionnaire, which represents the extent to which general or long-term goals or objectives have not been well-defined by the team leader.

Transformational leaders motivate their co-workers through a vision, and intellectual stimulation means transformational leaders tell their followers rather what to achieve than how to do the job. Both should reduce unclarity of goals in the team. Provided with a general vision and long-term objectives, the team may achieve a higher level of coordination and emergence, increasing its effectiveness. Thus, we assumed unclarity of goals to be negatively related to team effectiveness.

*H3. Unclarity of goals will partially mediate the relationship between transformational leadership and team effectiveness, with higher scores in transformational leadership associated to reduced unclarity of goals and thus to greater team effectiveness*.

**Figure 1:**
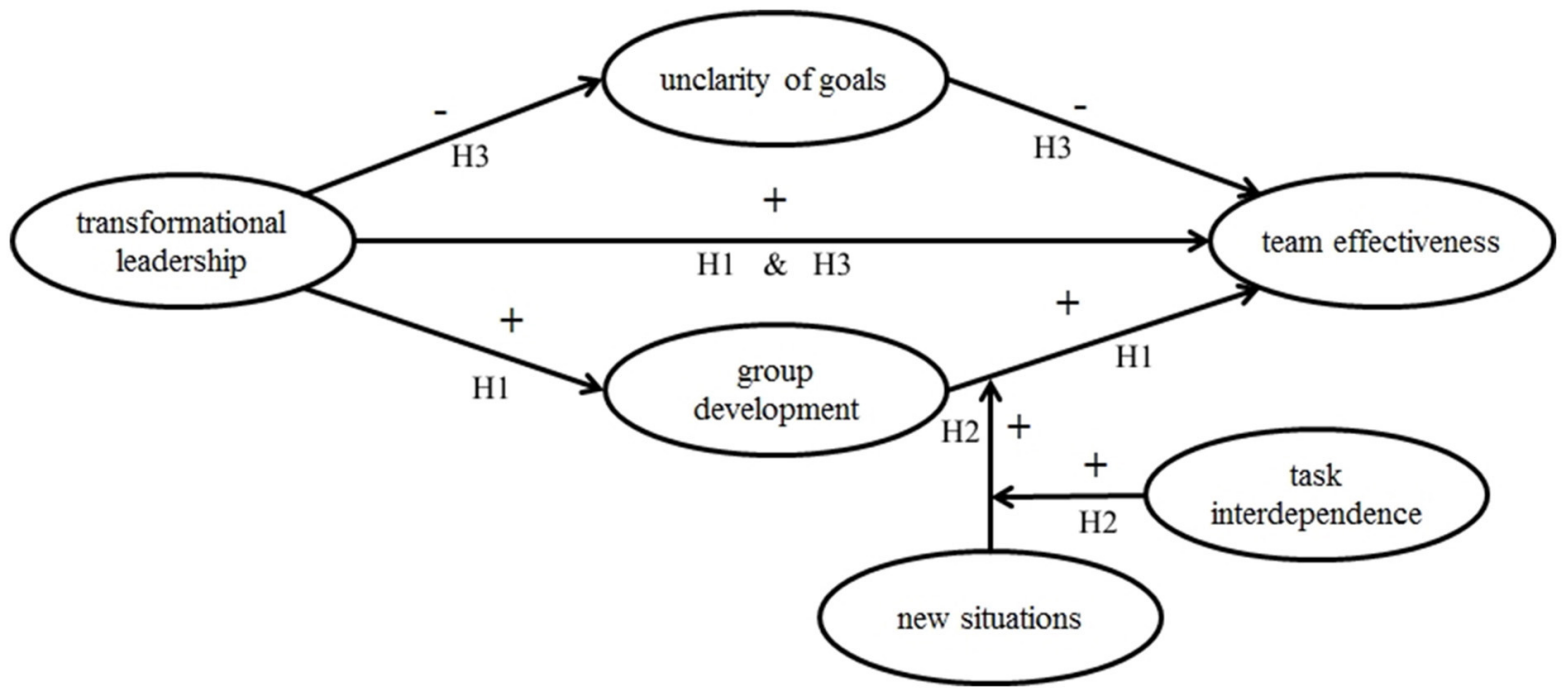
Model 1, representing the hypotheses H1, H2, and H3.

Figure 1 gives an overview of model 1.

Hypotheses H1, H2, and H3 formed model 1. However, following Weiss and Hoegl (2016), new situations could also increase the team's need to adapt and thus decrease its efficiency. Sicotte and Bourgault (2008) found that fuzziness, which resembles the here used variable new situations, correlated negatively to measures of performance. When a team scores high on new situations, then the team needs to adapt. The adaptation process will consume time and resources (Weiss and Hoegl, 2016), thus temporarily lowering performance. The more often a team needs to adapt, the lower its efficiency will be. New situations may also be detrimental to the team members' motivation, in case that the adaptation renders previously done work useless: the expected reward for previous efforts is suddenly removed. This justifies an alternative hypothesis that introduces new situations as a factor that has a direct influence on team performance.

Furthermore, new situations is a subjective measurement. Independently of the true amount of changing demands, the team's appraisal may protect it from the respective negative consequences. Transformational leaders who motivate team members through a long-term vision may be able to buffer the supposed decrease in motivation that could result from frequently adapting project plans to changing outside demands. Intellectual stimulation and individualized consideration could further increase the team members' abilities to deal with disruptions quickly and thus perceive them as less disturbing. A transformational leader's individually considerate behaviors could empower team members (Dionne et al., 2004). While research results at team level are still missing, Maynard et al. (2015) suspect empowerment to foster team adaptation and propose to further research this topic.

We assumed that transformational leadership could lead to a decrease in the measurement value of new situations, which in turn would correlate negatively with team effectiveness. Thus, new situations was also eligible as a mediator, and we created an alternative model 2 based on hypotheses H1 and H3 and substituting H2 by H2a.

*H2a. New situations will partially mediate the relationship between transformational leadership and team effectiveness, with higher scores in transformational leadership being associated to a lower score in new situations and thus to greater team effectiveness*.

Adapting to a new situation requires behavioral changes (Pulakos et al., 2002). We assumed that clearly defined interdependencies among the team members would speed up the adaptation process. If interdependence is low, then the number of options is high, e.g., everybody might be eligible for a new task. If, however, a task needs to be fit into a neatly organized set of interdependencies, then the available options are limited and the decision will be made faster, which saves resources. Additionally, we assumed that teams in which work was organized in a way that required team members to frequently exchange outputs among each other, adaptation would be easier to achieve. So, in teams experiencing new situations, we expected task interdependence to dampen the negative impact of uncertainty on team effectiveness.

Therefore, assuming H2a to be true, we expected the structure of the team's work, as represented by task interdependence, to moderate the effect of task uncertainty.

*H4. Task interdependence will moderate the relationship between new situations and team effectiveness as stated in H2a, with greater task interdependence associated to a weaker relationship between new situations and team effectiveness*.

**Figure 2:**
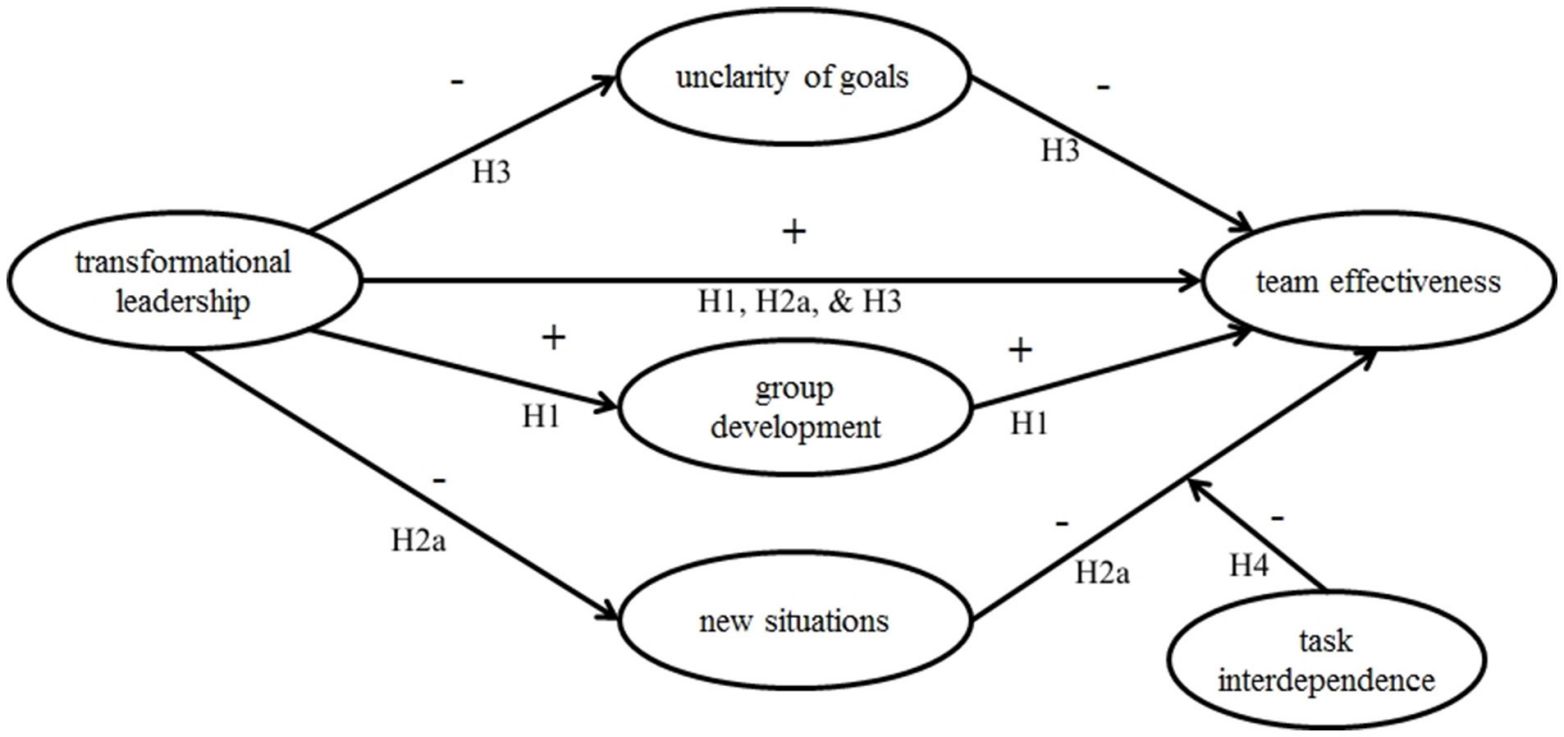
Model 2, representing the hypotheses H1, H2a, H3, and H4.

Figure 2 depicts model 2.

### Topic delimitation: uncertainty avoidance

Another variable that may determine how teams deal with uncertainty is uncertainty avoidance, e.g., as measured by the Uncertainty Avoidance Index (UAI, Hofstede et al., 2010). Its origins are in cross-cultural psychology and the following paragraphs explain why it was not included in our model.

Uncertainty avoidance is “the extent to which the members of a culture feel threatened by ambiguous or unknown situations. This feeling is […] expressed through nervous stress and a need for predictability” (Hofstede et al., 2010, p. 191). Some researchers have argued that high uncertainty avoidance will hamper innovation (Shane, 1993). However, with regard to this assumption, research has produced contradictory outcomes (Hofstede et al., 2010, pp. 211): Studies at national level have either found a negative relationship between uncertainty avoidance and innovation (Shane, 1993), or no relationship at all (Rinne et al., 2012). Hofstede et al. (2010) argued that cultures with low uncertainty avoidance excelled at producing new ideas, while cultures with high uncertainty avoidance were better at implementing such ideas into new processes or products.

This is interesting in the sense that depending on national culture, teams or individuals may apply different strategies to cope with uncertainty, which may in turn have an impact on performance. However, as the here-presented study is based on a sample from one national culture and from one organization, we did not include uncertainty avoidance into our model. If any effects exist, they will rather affect the international interpretability of the model.

Additionally to studies at national level, Hofstede's UAI can also measure individual differences: Zhang and Zhou (2014) found that in followers with high uncertainty avoidance, empowering leadership is related to higher creativity—but only if they trust their superior. This finding is likely to apply to transformational leaders, as they are expected to empower followers through intellectual stimulation (Bass et al., 2003). However, we planned to test our model at group level, we refrained from including individual level variables. Despite an individual's preference for avoiding or embracing uncertainty, different types of task uncertainty may have different effects in teams of knowledge workers. Such possible differences between sub-types of task uncertainty have been disregarded in previous research (e.g., Faraj and Yan, 2009; Gardner et al., 2012). From the perspective of cross-cultural psychology, knowing the effects of different types of uncertainty on work processes or outcomes may also aid in resolving the above mentioned dispute.

## Materials and methods

### Participants and procedure

Five hundred and one team members from 226 teams and 104 team leaders from a German research organization completed an online-questionnaire (Table 1). Submitting the questionnaire required answering all items. Thus, there were not any empty fields in the data matrix. Each team had at least three members, in addition to the leader. Mean age was 34.3 years (*SD* = 11.8). 32.9% had worked 2 years or less on their team, 32.5% between 2 and 5 years, and 31.5% more than 5 years. The study design was approved by the organization's workers' council (German: Gesamtbetriebsrat). Section Data Aggregation describes the data aggregation that resulted in (1) the final sample of 107 teams, composed by data from the team members, and (2) a sample of 54 of these teams, in which measurements of team effectiveness were provided by the leaders. We used the first sample for testing the model and the second sample to check for common source bias.

**Table 1:** Sample description.

	**All team members (*****N*** = **501)**		**107 selected teams (*****N*** = **408)**	
	***N***	**per cent (%)**	***N***	**per cent (%)**
Male participants	343	68.5	277	67.9
Female participants	158	31.5	131	32.1
Job: Researcher	423	84.4	346	84.8
Job: Administration	42	9.6	34	8.3
Job: Facility Management / Workshop	23	4.6	17	4.2
Job: IT-Services / PR-Services	13	2.6	11	2.7
0-2 years on the team	165	32.9	133	32.6
2-5 years on the team	178	35.5	144	35.3
5+ years on the team	158	31.5	130	31.9

*N, Number of individuals*.

### Measures

#### Transformational leadership

Most research on transformational leadership relied on the MLQ (Bass and Avolio, 1995). Yet its dimensionality has been questioned (Bycio et al., 1995), and Berger et al. (2012) showed that transformational leadership can be measured as a unidimensional construct. Thus, we used the German version of the HSA-TFL short-scale (8 items, Cronbach's α = 0.93). The instrument had previously been validated successfully in a German sample by Berger and Guàrdia. Example item of the follower questionnaire: (“Ich vertraue auf seine/ihre Fähigkeiten, Hindernisse jeder Art zu überwinden.” (“I have trust in his/her ability to overcome any obstacle”).

#### Group development

We used the German translation of the group development questionnaire based on Navarro et al. (2015), which we had previously validated in a German sample. In the validation study, the unidimensional structure was confirmed and the internal consistency was good (8 items, Cronbach's α = 0.87). Example item: “In meiner Arbeitsgruppe teilen wir untereinander Instrumente, Ressourcen und Informationen” (“We share tools, resources, and information).”

#### Task uncertainty

We used the German version of the MITAG model (Navarro et al., 2011), as resulting from our previous validation study, to measure unclarity of goals (4 items, Cronbach's α = 0.78), and new situations (3 items, Cronbach's α = 0.68). Example item: “In meiner Arbeitsgruppe ist es für uns ganz klar was wir mit unserer Arbeit erreichen sollen” (“We are very clear on what we must achieve with our work”).

#### Task interdependence

We translated the seven items developed by Van der Vegt et al. (2001) into German, using a back-translation process to avoid translation errors based on cultural or linguistic differences (ITC, 2005). In our data, the internal consistency (Cronbach's α) of this one-dimensional measure was 0.76.

#### Team effectiveness

We translated the twelve-item effectiveness-scale presented by Navarro et al. (2011) into German, following a back-translation process (ITC, 2005). These items are based on the normative model proposed by Hackman (1987). The internal consistency (Cronbach's α) of this one-factorial measure was 0.89 in our sample. Example item: “In meiner Arbeitsgruppe arbeiten wir wirksam.” (“We are efficient performing our tasks”).

Task Interdependence was measured using a seven-point Likert-scale, whereas all other instruments were presented with a five-point Likert-scale.

### Data aggregation

Team members answered all the above named instruments, while the team leaders answered only the items measuring team effectiveness. The data collected from the team members were aggregated at team level.

The wide-spread use of an equal distribution for calculating r_wg_ or r_wg(j)_ has been criticized (LeBreton and Senter, 2008), and it has been argued that 0.70 may be inadequate as a cut-off value for r_wg_ or r_wg(j)_ (Biemann et al., 2012). For ICC(1) and ICC(2), commonly accepted cut-off values do not exist, either. Researchers are recommended to calculate different indicators, e.g., r_wg(j)_, ICC(1) and ICC(2), to carefully pick null distributions, and to consider the level of agreement expected or required for the specific type of data, in comparison to other research in the area (Biemann et al., 2012). Based on these recommendations, we chose the following approach.

First, we calculated team means if at least two measurements were available from the same team, resulting in a sample of 133 teams (408 individuals). Mean age in this reduced sample was 34.3 years (*SD* = 11.9). Then, we calculated r_wg(j)_ (Bliese, 2000) to delete the groups with the lowest agreement. While Biemann et al. (2012) recommend not deleting groups with low agreement, in favor of test power, we considered deleting such teams and thus sacrificing test power as the more conservative approach. Despite the known criticism (LeBreton and Senter, 2008), in this case using an equal distribution was justified by three reasons: (1), we only used r_wg(j)_ for comparisons among teams, which means that any bias introduced by a potentially inadequate null distribution would affect all teams equally; (2) none of the restrictions mentioned by Meyer et al. (2014) seemed applicable to our data and thus no other distribution was more favorable, and (3) the null distribution was frequently used in recent leadership research (Biemann et al., 2012), which increases the comparability among studies. We deleted 26 teams in which either one r_wg(j)_ value was below 0.40, or in which four r_wg(j)_ values were below 0.70. The latter cut-off was chosen as, despite the mentioned criticism, it is the most commonly used limit (Biemann et al., 2012); the former was chosen at will. The coefficients resulting after eliminating 26 teams are shown in Table 2.

**Table 2:** Intra-group agreement measures of 107 teams to undergo further analysis.

**Measure**	**Number of items**	**Mean r_wg(j)_**	**ICC(1)**	**ICC(2)**
Unclarity of goals	4	0.83	0.36	0.64
New situations	3	0.79	0.13	0.64
Group development	8	0.80	0.17	0.50
Team effectiveness	12	0.83	0.23	0.38
Task interdependence	7	0.78	0.35	0.49
Transformational leadership	8	0.88	0.19	0.63

*Mean r_wg(j)_ is the arithmetic mean of the r_wg(j)_ score, a within-group interrater agreement, over 107 teams (Bliese, 2000). ICC1 and ICC2 are the Intra-Class-Correlation Coefficients 1 and 2 (Bliese, 2000)*.

In the resulting sample of 107 teams, we calculated ICC(1) and ICC(2) (Bliese, 2000). We required ICC(1) to be above 0.10 and ICC(2) to be above 0.30. These values correspond to the indices obtained in other leadership studies (Biemann et al., 2012). As these criteria were met (Table 2), we assumed that in the remaining sample, the aggregation was adequate.

### Datasets and missing data

We tested all hypotheses using the sample of 107 teams in which team effectiveness measures were provided by the team members. There were not any empty cells in the final dataset, as participants could only return completely answered questionnaires, and as the 26 teams with low agreement were fully removed during the aggregation process. Procedures for dealing with missing data were thus unnecessary. In 54 of the 133 aggregated teams, a measurement of team effectiveness by the team leader was available. Thus, by replacing the effectiveness measure from the members by that obtained from the leaders, we obtained a second dataset of 54 teams. We used this second sample to check for common source bias.

### Analysis of data

We used IBM SPSS Amos version 22 for structural equations modeling (SEM). We chose SEM for hypothesis testing (all hypotheses: models 1 and 2) for its benefit of correcting for measurement errors through the use of latent variables (Preacher and Hayes, 2008).

At individual level, we conducted separate confirmatory factor analyses (CFA) on the task interdependence measure and the team effectiveness measure. To assess the impact of common source variance, we applied Harman's test of common method bias (Podsakoff et al., 2003). For the same purpose, we additionally substituted the team members' measures of team effectiveness by their leaders' judgements of team effectiveness and conducted a regression analysis on the resulting sample of 54 teams, using the PROCESS macro for mediation effects (H1), version 2.13 (Hayes, 2015), and hierarchical regression analysis for moderation effects (H4).

We tested for the requirements of mediation (Baron and Kenny, 1986), and examined the significance of the indirect effect and the single predictors using the Amos 22 BC-bootstrapping procedure (Preacher and Hayes, 2008). Moderation (H2 and H4) was tested by including latent interaction variables. We followed the approach proposed by Marsh et al. (2004), and the additional recommendations by Foldnes and Hagtvet (2014). We used the following cut-off-criteria for the SEM: RMSEA (<0.08), based on MacCallum et al. (1996) and χ^2^/*df* (<5), based on Schumacker and Lomax (2004). In CFA, we additionally required TLI (>0.95), following Hu and Bentler (1999). For model comparison, we used χ^2^/*df* and RMSEA. For hypothesis testing, we set the Type I error at α = 0.05.

## Discussion

### Confirmatory factor analysis

**Figure 3:**
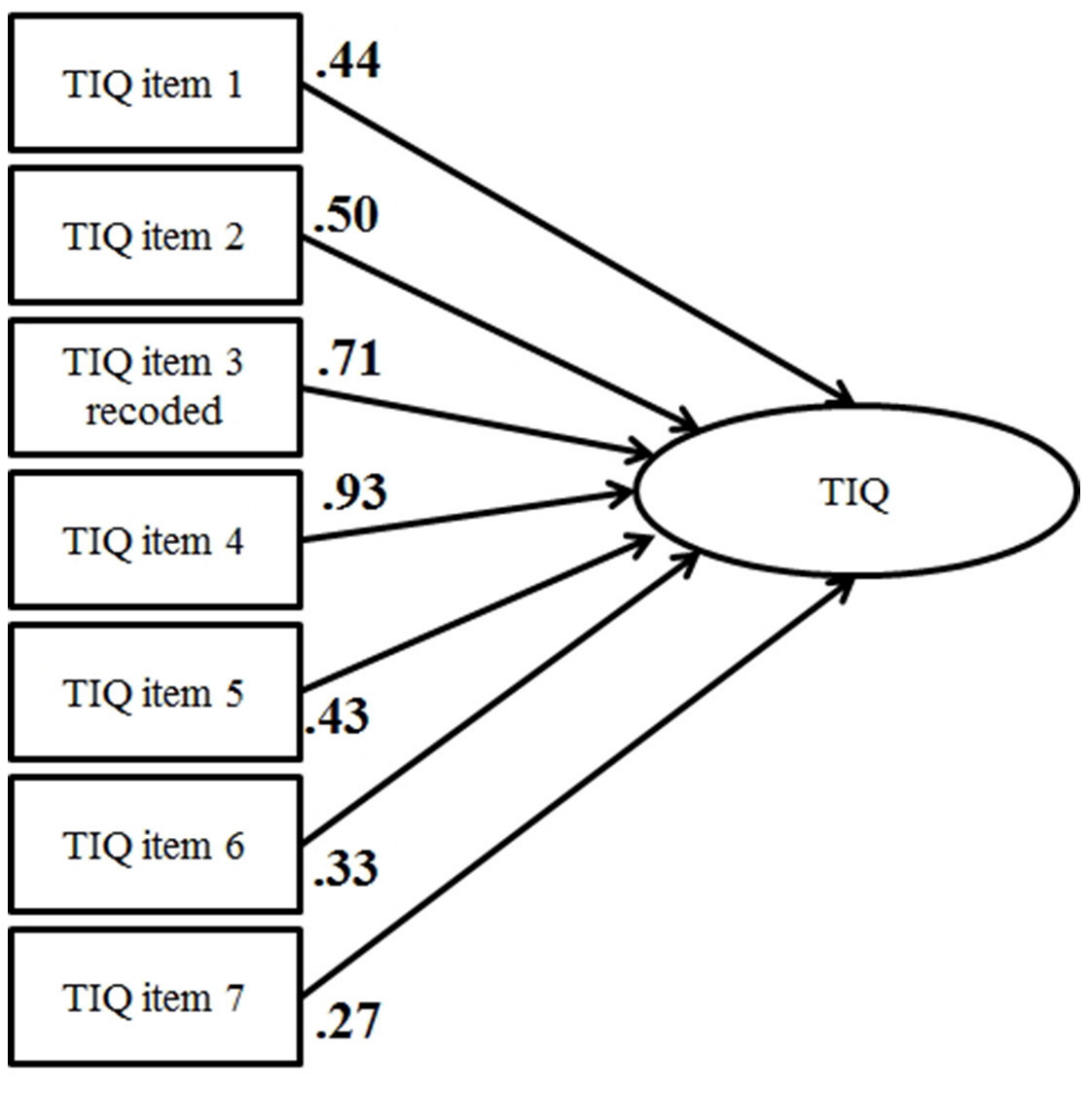
CFA of the Task Interdependence Questionnaire (standardized coefficients).

**Figure 4:**
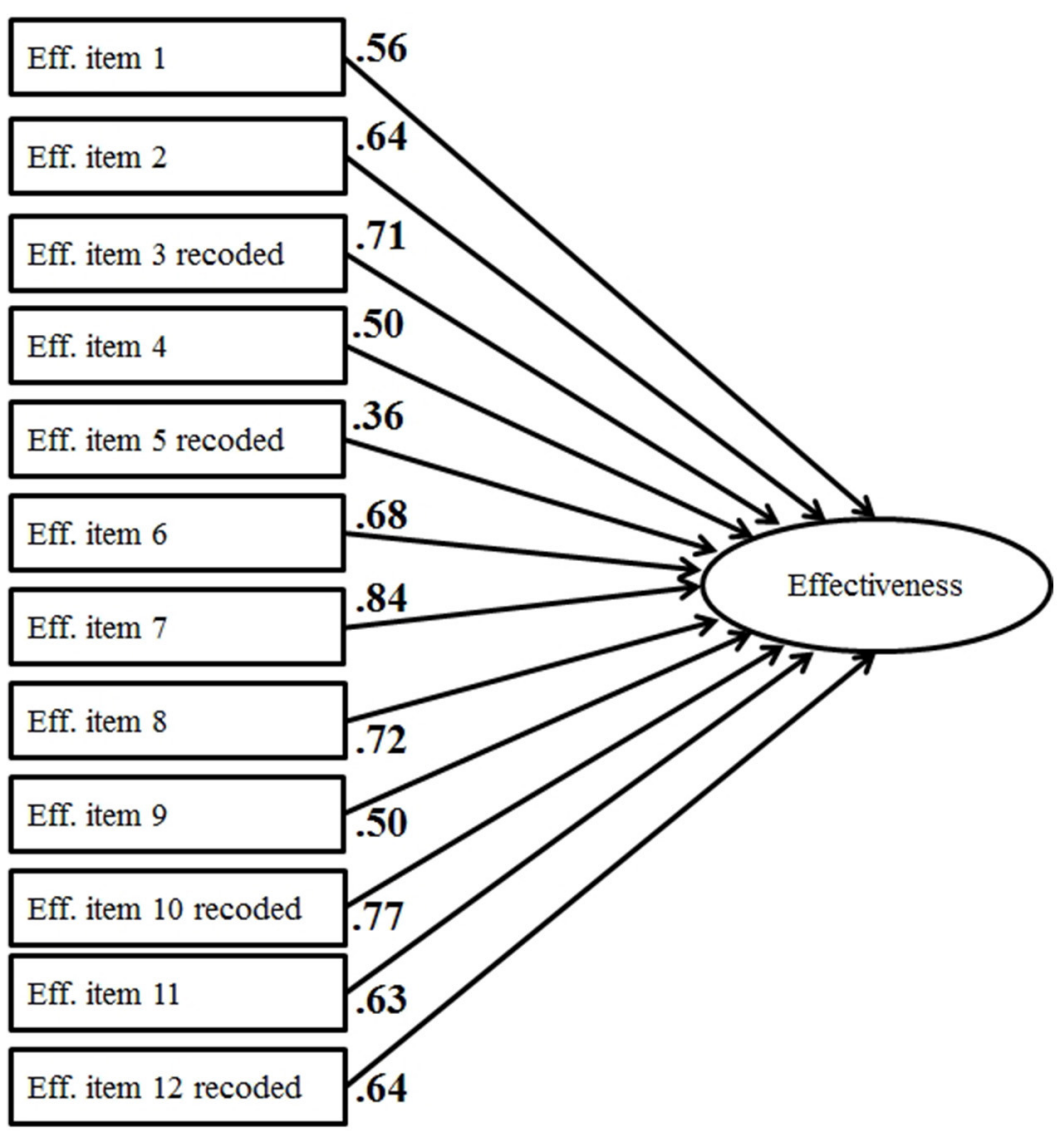
CFA of the Team Effectiveness measure (standardized coefficients).

Both translated instruments, the task interdependence questionnaire (Figure 3) and the team effectiveness instrument (Figure 4), proved to be one-factorial (Table 3).

**Table 3:** Model fit parameters.

**Model**	**χ^2^**	***df***	**χ^2^/*df***	***p* (χ^2^)**	**TLI**	**RMSEA**
CFA—task interdependence	25.92	11	2.36	0.000	0.97	0.05
CFA—team effectiveness	96.31	38	2.53	0.000	0.96	0.06
Model 1	649.28	422	1.54	0.000	0.89	0.07
Model 2	1016.52	655	1.55	0.000	0.85	0.07

*χ^2^ is the Chi-Square represented by CMIN in Amos 22, and df is the respective number of degrees of freedom. p (χ^2^) is the significance level of the χ^2^ statistic, named P in Amos 22. TLI, Tucker-Lewis Index; RMSEA, Root Mean Square Error of Approximation (Arbuckle, 2013)*.

### Testing model 1

H1 was confirmed. The preconditions of mediation (Baron and Kenny, 1986) were fulfilled, as transformational leadership predicted team effectiveness significantly by c = 0.78 (standardized coefficient; *p* < 0.001) when no mediator was present. With group development present as mediator, this relationship dropped to c' = 0.14, while a = 0.73 (*p* < 0.001) and b = 0.87 (*p* < 0.001). The total interaction effect of transformational leadership on effectiveness was significant at *p* < 0.01 after BC-Bootstrapping (2-tailed).

H3 was rejected, as unclarity of goals showed a low and statistically insignificant relationship to team effectiveness. Transformational leadership was negatively related to unclarity of goals (*p* < 0.01).

Allowing for the residuals of group development and unclarity of goals to covary, as recommended by Preacher and Hayes (2008), did not alter the reported results: changes in standardized parameters were less or equal 0.01.

The model including the latent interaction variable for testing H2 would not converge, due to discrepancies between product indicators. Therefore, we abandoned H2 and tested the alternative model 2.

### Testing model 2

H2a was confirmed: with new situations present as a sole mediator, the effect of transformational leadership on team effectiveness dropped to c' = 0.66 (*p* < 0.05), while a = −0.50 (*p* < 0.05) and b = −0.29 (*p* < 0.05). With GD and unclarity of goals present (model 2 in Table 3), the positive relationships were still significant (*p* < 0.05). The total interaction effect of transformational leadership on effectiveness was significant (*p* < 0.05) after BC-Bootstrapping (2-tailed). However, adding new situations as a third mediator did not further decrease the direct effect of transformational leadership on team effectiveness.

H4 was confirmed in the SEM with the estimate for the effect of the latent interaction variable on team effectiveness at 0.13 (*p* < 0.05): when task interdependence was high, the negative relationship between new situations and team effectiveness was weaker. Task interdependence was not a predictor of team effectiveness (b = 0.04, *p* > 0.05).

**Figure 5:**
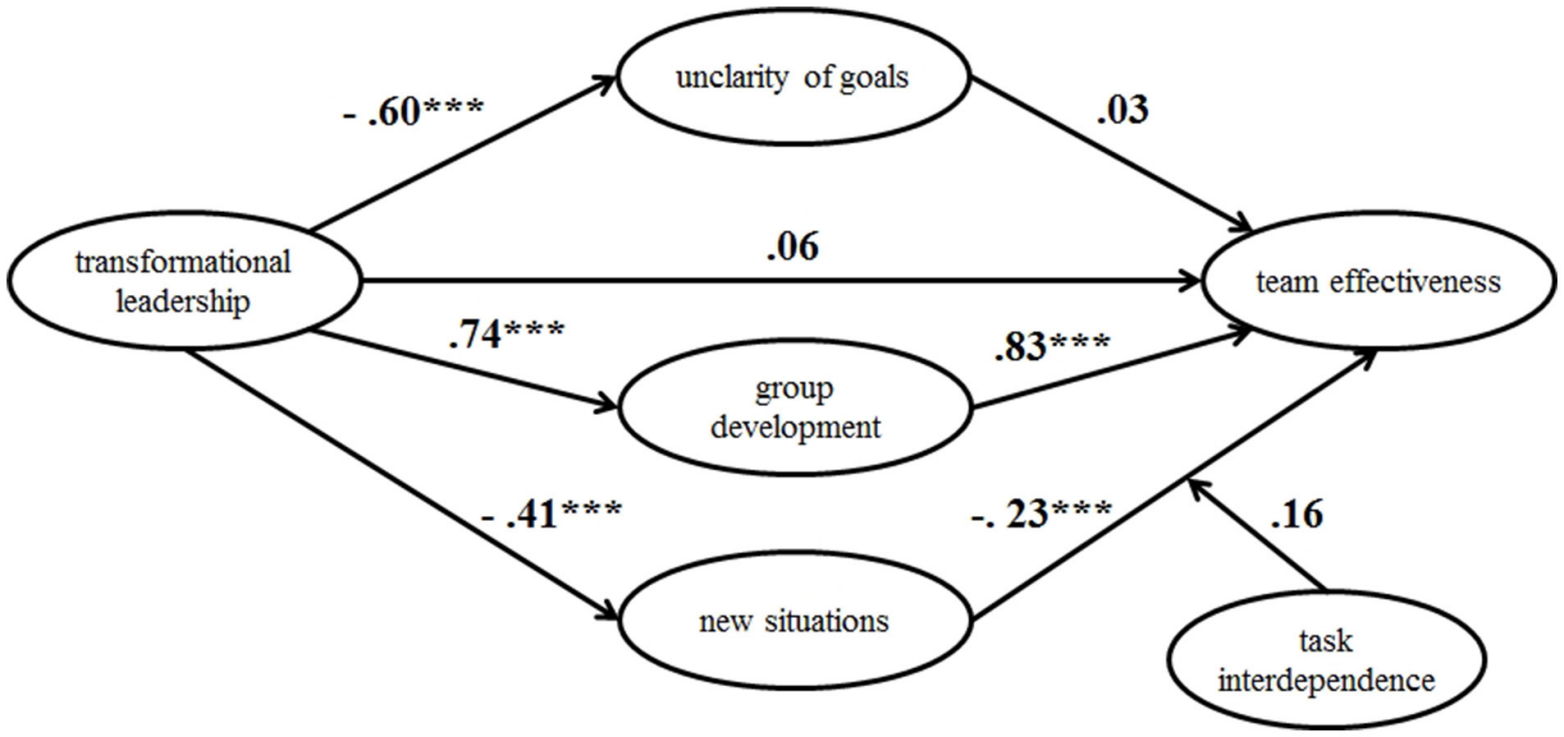
Structural equation model 2 with standardized estimates. ^***^p < 0.001.

Comparing this model 2 to model 1 (Table 3) is difficult, as it contains two additional variables (new situations and task interdependence). However, with respect to χ^2^/*df* and RMSEA, the loss of fit is minimal. Thus, model 2 can be accepted. Figure 5 summarizes the identified relationships.

### Assessment of common method bias

Harman's single factor test identified a factor that accounted for 36.8% of the entire variance of the variables: unclarity of goals, new situations, group development, team effectiveness, and transformational leadership. The regression analysis conducted with the sample of 54 teams that contained leader data confirmed the indirect effect of group development, with the 95% CI between 0.19 and 0.56. As a consequence, the identified relationships would remain relevant after correcting for a possible common method bias. The moderation effect of task interdependence could not be confirmed in a hierarchical regression analysis using team effectiveness measures from leaders. In Table 4, we provide the Pearson-correlation coefficients between the mean scores of the variables in the model.

**Table 4:** Pearson correlations of mean scores.

		**1**	**2**	**3**	**4**	**5**	**6**
1	Transf. leadership	(0.93)					
2	GD	0.64^**^	(0.87)				
3	MITAG (new sit.)	−0.50^**^	−0.51^**^	(0.68)			
4	MITAG (unclar. goals)	−0.32^**^	−0.33^**^	0.65^**^	(0.78)		
5	Task interdependence	0.72^**^	0.86^**^	−0.57^**^	−0.50^**^	(0.76)	
6	Team effectiveness	0.37^**^	0.47^**^	−0.23^*^	−0.03	0.40^**^	(0.89)

*N = 107 teams*.

** *Indicates significance at p < 0.01*.

* *Indicates significance at p < 0.05. The main diagonal contains Cronbach's α*.

## Findings

### Main findings

This work made three main contributions to the state of the art in leadership research: (1) it was, as far as we know, the first to investigate the role of leadership in the context of knowledge-work, taking task characteristics (i.e., uncertainty and interdependence) into account; (2) with group development and task interdependence, it considered both, interpersonal and structural coordination mechanisms; and (3) it addressed methodological limitations of previous research, such as unspecific measurements of uncertainty, issues related to the construct of group cohesion, and the restriction to very homogeneous samples.

The main finding is that group development mediates the positive relationship between transformational leadership and team effectiveness. Transformational leaders do not just create cohesion, some sort of social attraction, in the team. They achieve higher acceptance of and identification with group goals through visionary leadership, and their team members develop better interpersonal relationships among each other, which leads to improved sharing of resources and better coordination. This is why individual consideration and intellectual stimulation pay off beyond performance improvements at the individual follower level.

The results also show that task uncertainty (i.e., new situations) is indeed a relevant phenomenon, as it affected all groups of participants, to a greater extent than expected. New situations, the task uncertainty factor relating to unstable environmental conditions or unpredictably changing outside demands, is *per-se* detrimental to team effectiveness, as the team's efforts to adjust consume additional resources. Teams led by transformational leaders report to suffer less from such unstable conditions and in turn show higher effectiveness. This mediation-effect of the factor new situations does not explain any additional variance compared to the mediator group development. Thus, the data shows that it is by fostering teamwork (i.e., developing the team better) and creating emergence among team members, that transformational leaders achieve better team performance. The reduced task uncertainty with respect to new situations is rather a byproduct of this effect.

Furthermore, the data indicates that the structural coordination mechanism of task interdependence may help teams become less affected by such unstable environmental conditions: the negative effect of unstable environments on team effectiveness was lower when task interdependence was high. This means that beyond the improved sharing of resources among team members, which results from improved group development, the way work is organized can have an additional effect. Supposedly, teams adapt easier to new situations if cooperation mechanisms are well-defined.

Despite these mediating effects and contrary to what some authors have suggested, we did not find any evidence of task uncertainty (i.e., new situations) moderating the influence of transformational leadership on team effectiveness. Also, the mediating role of the task uncertainty factor unclarity of goals was not confirmed. The data shows that transformational leaders, by definition expected to motivate team members with a vision, reduce unclarity of goals in their teams. Nevertheless, this did not positively affect team effectiveness. In research and development, unclear objectives may diminish efficiency but they also allow for innovation. This is in line with other findings. Eisenbeiß (2009), for example, reported that although transformational leadership had a positive effect on follower creativity, it also increased the followers' dependence on the leader, which in turn had a negative impact on creativity.

### Theoretical implications

Apart from the described findings, this research has further theoretical implications. We did not identify any moderating effect of task uncertainty, as we assumed based on previous literature (Bass and Riggio, 2006; Frost et al., 2010). Some research questions came up: (1) subsequent studies could investigate whether another here not represented aspect of task uncertainty fits the described role as a moderator; (2) researching the role of the team members' appraisal of uncertainty may provide helpful insights; and (3) studies to be conducted in other cultures could test the generalizability of the results, e.g., across different levels of uncertainty avoidance. Of particular interest to researchers might be the finding that different types of task uncertainty, such as new situations and unclarity of goals, may play different roles in teams of knowledge workers. This is a first step toward refining existing models that include effects of uncertainty, and toward specifying uncertainty aspects more precisely in future studies, e.g., in research on team adaptation (see Maynard et al., 2015).

### Practical implications

The results indicate that organizations should foster transformational leadership and remove barriers that may hinder group development. Task interdependence among team members, which is sometimes avoided as a possible source of problems, may also have positive effects on how the team deals with uncertainty. Many teams in non-research jobs reported task uncertainty to be higher than we had expected. For practitioners, this highlights the importance of group development and transformational leadership in a broad spectrum of jobs.

### Limitations and implications for research

This study has several limitations. First, it was cross-sectional and non-experimental. Thus, our design does not allow for causal interpretation. Following an experimental design was impossible, as we could not manipulate transformational leadership long enough for groups to develop significantly. If transformational leadership and group development are more stable over time than team effectiveness, then the indirect effect may have been overestimated (Maxwell and Cole, 2007). However, the causal effect of transformational leadership on team effectiveness has already been demonstrated experimentally (Avolio et al., 2009), and was replicated here. Assuming a reciprocal relationship between transformational leadership and group development seems difficult to justify on a theoretical level, such as a reciprocal relationship between transformational leadership and team effectiveness. However, Mullen and Copper (1994) argued that the relationship between cohesion and team effectiveness is reciprocal, with a stronger causal effect of the group process on the outcomes. The same may apply to the relationship between team effectiveness and group development: team success could, for example, foster identification with the team.

Second, our sample contained few responses from teams with low task interdependence or low task uncertainty. This may, additionally to the mediation effects identified, have obscured potentially existing moderation effects of task uncertainty. The findings thus represent R&D teams with rather high task uncertainty.

Third, for reasons of model complexity, it was not possible to take into account to which extent the team members worked on projects together with their teammates or in virtual teams outside the official team structure. To overcome this limitation, we recommend researching the extent to which resources provided by the core team can be carried over into the work on virtual teams, or limiting a future studies to a context in which team members are not participating in virtual teams.

Fourth, while the mediation effects identified were maintained when checking for common method bias, the moderation effect of task interdependence was not. Thus, this result has to be interpreted with caution. For future work, we recommend collecting external outcome indicators, such as financial figures, to reduce potential single source bias.

Fifth, the sample was unbalanced toward researchers and male participants, which was due to the true distribution of genders (34% were women) and jobs (55% were researchers) in the organization (based on HR data from the year 2014). Our data correctly represent today's R&D sector with its limited gender diversity. Additionally, our sample was collected in only one organization and only in Germany; therefore, possible cultural influences, such as effects caused by the level of uncertainty avoidance, may lead to different results in other cultures.

With respect to future research, we also recommend exploring possible suppressor-effects on the relationship between unclarity of goals and team effectiveness. Data should be collected from samples with greater variability in task uncertainty and task interdependence. The findings may also be relevant for cross-cultural psychology: different types of task uncertainty have a different impact in the model. Researching the effects of uncertainty avoidance may thus require measuring the type of uncertainty faced by the participants.

We recommend the GD instrument for research, as well as for practical application in organizations; although caution is advised when comparing regression coefficients across studies, the strength of the identified relationships justifies this choice.

## Summary

In summary, task uncertainty affects a broad range of jobs in modern organizations, beyond the R&D area. Transformational leadership fosters group development and thus leads to greater team effectiveness. This goes along with turbulent situations being perceived less uncertain by team members. Task interdependence further buffers the negative effect of turbulent situations on team effectiveness.

## Ethics statement

This study was carried out in accordance with the recommendations of the Ethical Principles of Psychologists and Code of Conduct (incl. the amendment from 2010) by the American Psychological Association's (APA). All participants gave written informed consent in accordance with the Declaration of Helsinki. The protocol was approved by the workers' council of the participating organization.

## Author contributions

All authors (JL, RB, JN) fulfill the Frontiers requirements for authorship: (1) substantial contributions to the realization of the research, (2) drafting and revising the work for intellectual content, (3) approving the final version to be submitted, and (4) agreeing to be accountable for all aspects of the work and taking responsibility each on their own to ensure possible issues regarding accuracy or integrity are resolved. In detail, the contributions of the authors are the following: Definition of research objectives, models, and hypotheses: JL, RB, JN. Provision of materials (i.e., questionnaires): RB, JN. Data collection: JL. Data analysis plan: JL, RB, JN. Data analysis: JL. Principal article writing: JL. Article revision and proofreading: RB, JN. Final approval: JL, RB, JN.

### Conflict of interest statement

The authors declare that the research was conducted in the absence of any commercial or financial relationships that could be construed as a potential conflict of interest.
